# Usefulness of video laryngoscopy in tracheal intubation at thyroid surgical position for intraoperative neuromonitoring

**DOI:** 10.1038/s41598-024-55537-0

**Published:** 2024-02-29

**Authors:** Dongwook Won, Jung-Man Lee, Jiwon Lee, Young Jun Chai, Jin-Young Hwang, Tae Kyong Kim, Jee-Eun Chang, Hyerim Kim, Min Jong Kim, Seong-Won Min

**Affiliations:** 1 Department of Anesthesiology and Pain Medicine, Seoul National University College of Medicine, Seoul Metropolitan Government Seoul National University Boramae Medical Center, 20, Boramae-ro 5-gil, Dongjak-gu, Seoul, 07061 Republic of Korea; 2 Department of Anesthesiology and Pain Medicine, Anesthesia and Pain Research Institute, Yonsei University College of Medicine, Gangnam Severance Hospital, 211, Eonju-ro, Gangnam-gu, Seoul, Republic of Korea; 3 Department of Surgery, Seoul National University College of Medicine, Seoul Metropolitan Government Seoul National University Boramae Medical Center, Seoul, Republic of Korea; 4 Department of Anesthesiology and Pain Medicine, Seoul National University Hospital, Seoul, Republic of Korea

**Keywords:** Neural monitoring, Recurrent laryngeal nerve, Thyroid surgery, Vocal cord paralysis, Thyroid cancer, Nervous system, Health services

## Abstract

This observational study aimed to compare the glottic view between video and direct laryngoscopy for tracheal intubation in the surgical position for thyroid surgery with intraoperative neuromonitoring. Patients scheduled for elective thyroid surgery with intraoperative neuromonitoring were enrolled. After the induction of anesthesia, patients were positioned in the thyroid surgical posture with a standard inclined pillow under their head and back. An investigator assessed the glottic view using the percentage of glottic opening (POGO) scale and the modified Cormack–Lehane grade in direct laryngoscopy and then video laryngoscopy sequentially while using the same McGRATH™ MAC video laryngoscope at once, with or without external laryngeal manipulation, at the surgical position. A total of thirty-nine patients were participated in this study. Without external laryngeal manipulation, the POGO scale significantly improved during video laryngoscopy compared to direct laryngoscopy in the thyroid surgical position (60.0 ± 38.2% vs. 22.4 ± 23.8%; mean difference (MD) 37.6%, 95% confidence interval (CI) = [29.1, 46.0], *P* < 0.001). Additionally, with external laryngeal manipulation, the POGO scale showed a significant improvement during video laryngoscopy compared to direct laryngoscopy (84.6 ± 22.9% vs. 58.0 ± 36.3%; MD 26.7%, 95% CI = [18.4, 35.0] (*P* < 0.001). The superiority of video laryngoscopy was also observed for the modified Cormack–Lehane grade. In conclusion, video laryngoscopy with the McGRATH™ MAC video laryngoscope, when compared to direct laryngoscopy with it, improved the glottic view during tracheal intubation in the thyroid surgical position. This enhancement may potentially facilitate the proper placement of the electromyography tracheal tube and prevent tube displacement due to positional change for thyroid surgery.

## Introduction

Recurrent laryngeal nerve (RLN) injury stands as a significant complication of thyroid surgery, with reported incidences in up to 5.8% of thyroid surgeries^1^. Particularly, the incidence of permanent RLN injury escalates to 13–30% during thyroid cancer surgeries and secondary thyroidectomy^1^. Intraoperative neuromonitoring (IONM) utilizing an electromyography (EMG) tracheal tube aims to monitor the integrity of the RLN during thyroid surgery. For successful IONM, ensuring contact between the surface electrodes of the EMG tube and the true vocal cords is crucial^2^. Video laryngoscopes can facilitate optimal placement of the surface electrodes of the EMG tube^3^. However, the challenge of maintaining precise IONM persists during positional changes in the patient. To enhance thyroid gland exposure, patients are typically positioned in the thyroid surgical posture after tracheal intubation, involving neck hyperextension from the sniffing position with a roll under the neck and shoulders. This positional change to the thyroid surgical position can lead to tracheal tube displacement with varying degrees depending on the patient^4–6^. Therefore, re-verification of tracheal tube position may be necessary after assuming the surgical position^7^. To reduce repetitive laryngeal procedures while ensuring precise IONM during surgery, tracheal intubation with an EMG tube in the thyroid surgical position might be helpful^8–13^. However, two previous studies indicated a worsened glottic view during direct laryngoscopy in the surgical position for thyroid surgery, potentially complicating tracheal intubation^9,11^. Although a previous study presenting the possibility of standardized IONM in transoral endoscopic thyroidectomy, which was a new surgical approach technique for thyroidectomy, introduced a method of using a video laryngoscope after assuming the thyroid surgical position in thyroid surgery with IONM, usefulness of the video laryngoscope in thyroid surgical position was not quantitatively analyzed and presented^13^.

We hypothesized that the use of a video laryngoscope could be helpful in overcoming this issue during tracheal intubation in the surgical position for thyroid surgery. This study aimed to compare the glottic view between video laryngoscopy and direct laryngoscopy, when using the same McGRATH™ MAC video laryngoscope with a disposable Macintosh type blade, for tracheal intubation in the thyroid surgical position.

## Methods

**Figure 1:**
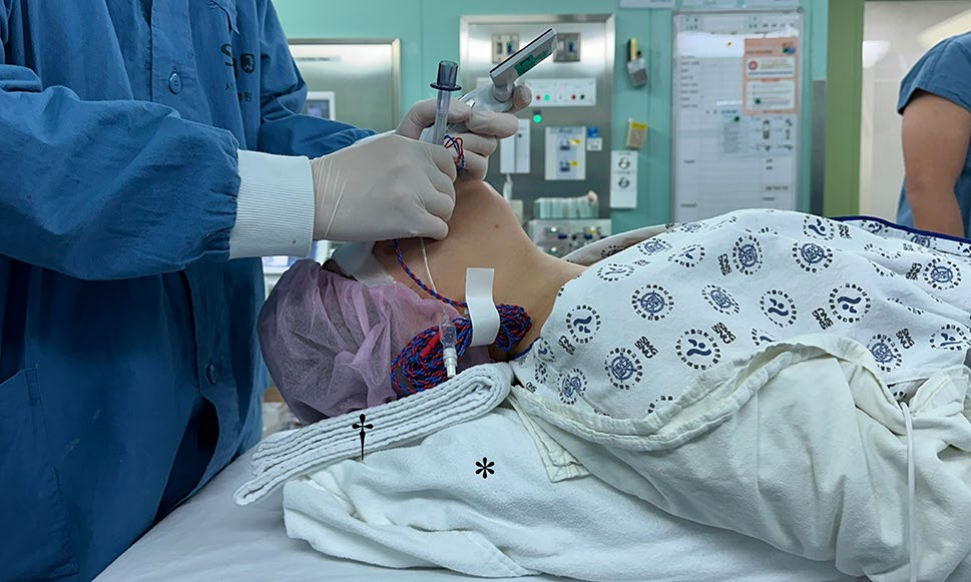
Prior to tracheal intubation, a standardized pillow (*) was placed beneath the head and back of the patient for the thyroid surgical position to prevent tube displacement, which is possible if the patient’s position should be adjusted to a thyroid surgical position after tracheal intubation without pre-positioning. If needed to prevent unnecessary hyperextension of patient neck, the thin clean towel (†) was applied. Permission was obtained from the patient.

This prospective observational study was approved by the Seoul Metropolitan Government Seoul National University Boramae Medical Center Institutional Review Board (IRB #20-2022-3) and written informed consent was obtained from all participants. Additionally, a written informed consent was obtained from one patient who underwent thyroidectomy with IONM to allow investigators to take a photo (Fig. 1) depicting tracheal intubation in the thyroid surgical position.

The trial was registered on ClinicalTrials.gov (NCT05251194, Principle investigator: Jung-Man Lee, https://clinicaltrials.gov/ct2/show/NCT05251194, Date of registration: 22 Feb 2022) prior to patient enrollment. The study protocol conformed to the ethical guidelines of the Declaration of Helsinki 2013. Reporting of the study adhered to the Strengthening the Reporting of Observational studies in Epidemiology (STROBE) guidelines. From March to May 2022, among patients scheduled for thyroidectomy, adult patients (≥ 19 years old) with American Society of Anesthesiologists (ASA) physical status classifications I–III were recruited. Patients anticipated to have difficult airways, such as severely limited neck extension or mouth opening, weak teeth, or a history of difficult airway, were excluded from the study. Demographic characteristics, including age, sex, height, weight, ASA physical status classification, and airway characteristics, including mouth opening, Mallampati classification, thyromental distance (TMD), neck length which is defined as distance between the sternal notch and thyroid prominence in the neutral head and neck position, and the neck circumference, were recorded.

The patient entered the operating room without premedication. Routine monitoring with electrocardiography, non-invasive blood pressure, and peripheral oxygen saturation were initiated. Anesthesia was induced with 1% lidocaine (30 mg), propofol (1.5 mg/kg), and remifentanil using a target-controlled infusion system with a Perfusor Space^®^ Syringe Pump (B Braun, Sheffield, United Kingdom). After neuromuscular blockade with rocuronium (0.6 mg/kg), Patients were positioned on an inclined pillow (Thyroid B-pillow, EMTAS, Seoul, Korea) (* in Fig. 1) which had a length of 50 cm, a height of 13 cm, and two slope angles of 30 (toward cephalad direction) and 20 (toward caudal direction) degrees from its bottom, with the upper body was slightly raised and the neck hyperextended^8^. If the patient neck was hyperextended unnecessarily, a surgeon adjusted the degree of neck extension with adding thin clean towels († in Fig. 1) under the patient head, so that the degree of neck extension could be minimized while ensuring adequate surgical approach for the surgery.

**Figure 2:**
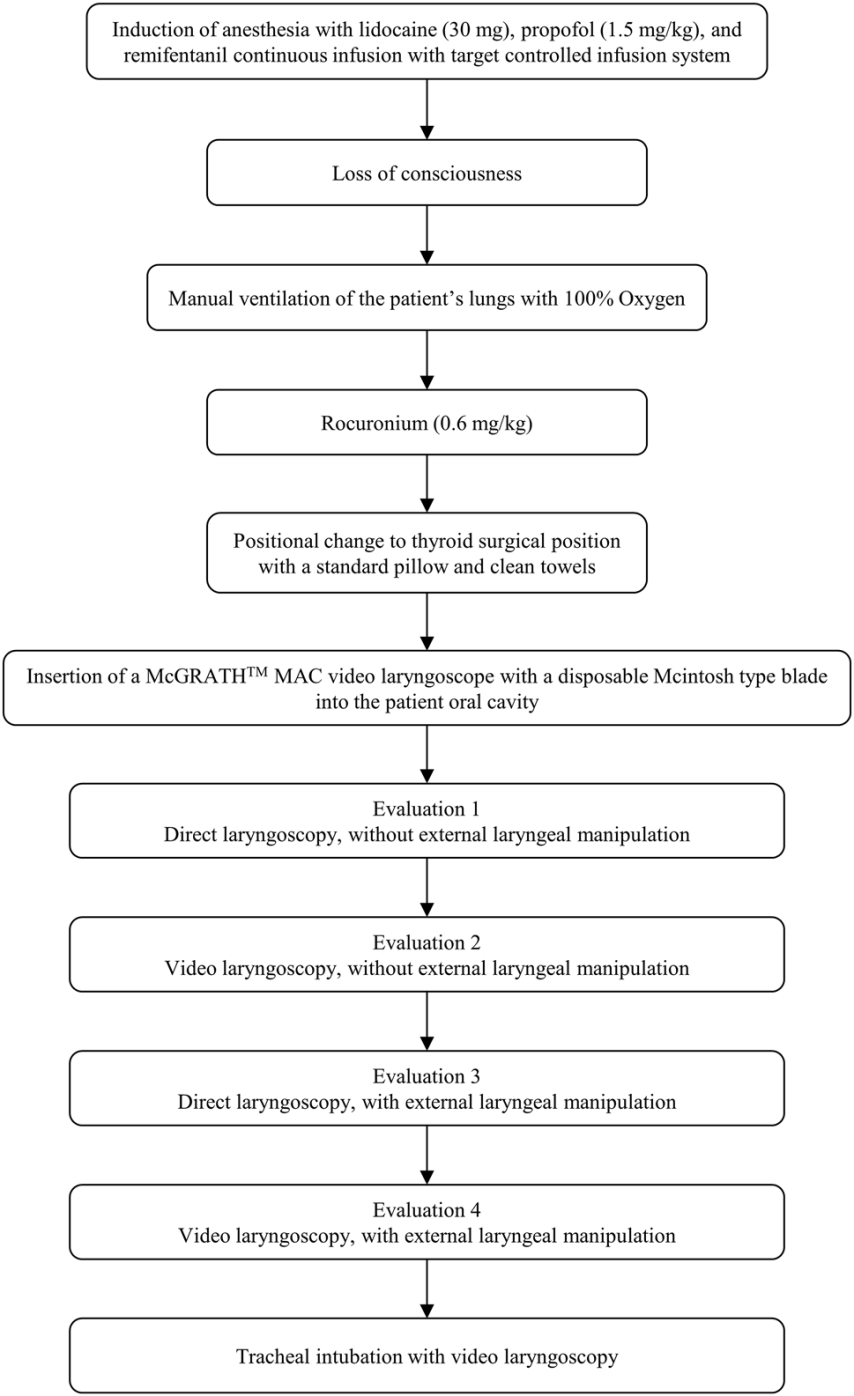
A flow diagram of the study protocol during the period between induction of anesthesia and tracheal intubation. Both direct and video laryngoscopies were evaluated sequentially, using the same McGRATH™ MAC video laryngoscope with a Mcintosh type blade at once. The modified Cormack–Lehane grade and percentage of glottic opening (POGO) scale were evaluated during laryngoscopies.

After positioning, the glottic views of the patient were evaluated using a video laryngoscope (McGRATH™ MAC, Aircraft Medical Ltd., Edinburgh, UK) with a disposable Macintosh type blade. The blade size was suited for patients (size 3 for females and 4 for males, usually). First, assuming that direct laryngoscopy was performed without seeing the monitor screen of the video laryngoscope, the glottic view was evaluated using the modified Cormack–Lehane (C–L) grade and percentage of glottic opening (POGO) scale without external laryngeal manipulation^14–16^. Subsequently, the glottic opening view was evaluated with both the modified C–L grade and POGO scale with video laryngoscopy while seeing the monitor screen of the video laryngoscope. Next, the glottic views in both direct laryngoscopy and video laryngoscopy were equally re-evaluated using the modified C–L grade and POGO scale with external laryngeal manipulation, such as individualized backward, upward, and rightward pressure (BURP) maneuver to obtain the best view in each patient (Fig. 2).

After evaluating the glottic views, tracheal intubation was performed using video laryngoscopy at the thyroid surgical position, trying to place the electrode of the NIM^®^ EMG endotracheal tube (Medtronic, FL, US) between the vocal cords (Fig. 1). Total intubation time was defined as the time from insertion of the blade of the video laryngoscope into the patient’s oral cavity to the confirmation of tracheal intubation by three consecutive waves of capnography on the patient monitor. Finally, the use of external laryngeal manipulation for real tracheal intubation and the difficulty of intubation with 4 grades of ‘easy/moderate/difficult/impossible’ was recorded.

The primary outcome was improvement of the POGO scale with video laryngoscopy compared to direct laryngoscopy, with and without external laryngeal manipulation, because exposing more true vocal cords might facilitate the accurate positioning of EMG tube and the POGO scale has good inter-physician/intra-physician reliability^15^. Secondary outcomes included an improvement in the modified C–L grade with video laryngoscopy compared to direct laryngoscopy, with and without external laryngeal manipulation to provide additional information on the glottic view that the POGO scale cannot provide as the modified C–L grade, especially 2B, 3, and 4 of it.

### Statistical analysis

Ordinal data are expressed as numbers (percentages) and tested using Wilcoxon signed-rank tests. Continuous data are expressed as mean ± standard deviation (SD) or median [interquartile range] according to the normality of the variables, which were tested using the Kolmogorov–Smirnov test. A paired *t*-test or Wilcoxon signed-rank test was used for analysis of continuous variables according to their normality. All statistical analyses were performed using SPSS Statistics 26.0 software (IBM Corporation, Chicago, IL, USA), and statistical significance was set at *P* < 0.05.

### Sample size calculation

In the literature review of this study, there were no previous studies on this issue. In two previous studies on thyroid surgery with IONM, the authors performed tracheal intubation with a direct laryngoscope at the thyroid surgical position and evaluated the modified C–L grade for the patients in those studies^9,11^. From the data of those studies, the modified C–L grade (1/2A/2B/3/4) in direct laryngoscopy was 11/48/61/19/5 at the thyroid surgical position. We assumed that the modified C–L grade 1/2A/2B/3/4 corresponds to 100%/50%/0%/0%/0% as the POGO scale, respectively. From this, we estimated the mean POGO scale in direct laryngoscopy at the thyroid surgical position to be approximately 20%. We hypothesized that the POGO scale would improve with a video laryngoscope by approximately 30% with a standard deviation of 35%. Therefore, the sample size was calculated to be 39, considering that the type I error rate was 0.01, and the type II error rate was 0.05, and the drop rate was 15%.

## Results

Thirty-nine patients were included in the study from March to May 2022. As none of the patients met the exclusion criteria, the data from all patients were used for statistical analyses. Patient characteristics are summarized in Table 1.

**Table 1:** Patient demographics and airway characteristics.

Characteristics	All (n = 39)
Sex (F/M)	28/11
Age (y)	45.7 ± 14.3
Height (cm)	162.8 ± 7.3
Weight (kg)	63.1 ± 12.1
Body mass index (kg/m^2^)	23.7 ± 3.8
American Society of Anesthesiologists physical status (1/2/3)	26/11/2
Mouth opening (cm)	4.6 ± 0.6
Mallampati score 1/2/3/4	12/13/9/5
Thyromental distance (cm)	7.2 ± 0.8
Neck length^a^ (cm)	7.5 ± 1.3
Neck circumference (cm)	35.2 ± 3.7

^a^Distance between the sternal notch and thyroid prominence in the neutral head and neck position. Data are presented as the mean ± standard deviation or number.

Without external laryngeal manipulation, the POGO scale was significantly higher in video laryngoscopy than in direct laryngoscopy at the thyroid surgical position (60.0 ± 38.2% vs. 22.4 ± 23.8%; mean difference (MD) 37.6%, 95% confidence interval (CI) = [29.1, 46.0], *P* < 0.001). The modified C–L grade (1/2A/2B/3/4) without external laryngeal manipulation was also improved in video laryngoscopy (n = 19/12/8/0/0) compared with direct laryngoscopy (n = 0/17/19/3/0) (*P* < 0.001) (Table 2).

**Table 2:** Comparison of the percentage of glottic opening (POGO) scale and modified Cormack–Lehane (C–L) grade between video laryngoscopy and direct laryngoscopy, without external laryngeal manipulation.

	Video laryngoscopy	Direct laryngoscopy	Mean difference (95% CI)	P-value
POGO scale (%)	60.0 ± 38.2	22.4 ± 23.8	37.6 (29.1, 46.0)	< 0.001^a^
Modified C–L grade (1/2A/2B/3/4) (n)	19/12/8/0/0	0/17/19/3/0		< 0.001^b^

Data are presented as the mean ± standard deviation or number (95% confidence interval). ^a^Paired *t*-test. ^b^Wilcoxon signed-rank test.

With external laryngeal manipulation, the POGO scale was significantly higher in video laryngoscopy than in direct laryngoscopy in the thyroid surgical position (84.6 ± 22.9% vs. 58.0 ± 36.3%; MD 26.7%, 95% CI = [18.4, 35.0] (*P* < 0.001). The modified C–L grade (1/2A/2B/3/4) with external laryngeal manipulation was improved in video laryngoscopy (n = 25/14/0/0/0), compared with direct laryngoscopy (n = 13/16/10/0/0) (*P* < 0.001) (Table 3). Finally, tracheal intubation was performed under video laryngoscopy without external laryngeal manipulation in 37 patients (94.9%), or with it in 2 patients (5.1%) to improve the glottic view. One of the 39 patients had difficulty in tracheal intubation under video laryngoscopy because the glottic view was recorded as 20% for the POGO scale in video laryngoscopy, even with external laryngeal manipulation.

**Table 3:** Comparison of the percentage of glottic opening (POGO) scale and modified Cormack–Lehane (C–L) grade between video laryngoscopy and direct laryngoscopy, without external laryngeal manipulation.

	Video laryngoscopy	Direct laryngoscopy	Mean difference (95% CI)	P-value
POGO scale (%)	84.6 ± 22.9	58.0 ± 36.3	26.7 (18.4, 35.0)	< 0.001^a^
modified C–L grade (1/2A/2B/3/4) (n)	25/14/0/0/0	13/16/10/0/0		< 0.001^b^

Data are presented as the mean ± standard deviation or number (95% confidence interval). ^a^Paired *t*-test. ^b^Wilcoxon signed-rank test.

The mean of total intubation time was 32.1 ± 6.1 s. Following the study protocol, anesthesia and surgery were performed in a routine manner at our institute. Surgery was successfully performed in all patients without repositioning of the EMG tube for IONM. There were no adverse events related to the study protocol, such as dental injury, hypoxemia, or aspiration.

## Discussion

In this study, our findings indicated a superior glottic view in video laryngoscopy with the McGRATH™ MAC video laryngoscope compared to that in direct laryngoscopy with it, whether performed with or without external laryngeal manipulation, during tracheal intubation in the thyroid surgical position for thyroid surgery with IONM. This was proven when evaluating the glottic view by the POGO scale and the modified C–L grade in the study. Based on these results, we believe that the combination of video laryngoscopy and the thyroid surgical position prior to tracheal intubation in thyroid surgery with IONM can improve the condition of IONM because proper placement of the EMG tube is easy by video laryngoscopy, and positional change of the EMG tube can be prevented concomitantly by pre-positioning the patient in the surgical position before tracheal intubation.

Video laryngoscopes are known to improve the glottic view in many circumstances. Consequently, video laryngoscopes have been used in several fields, including difficult airway management, pre-hospital intubation, education and training, and placement of special devices including double-lumen tubes, transesophageal probes, and EMG tube^17^. Correct placement of the EMG tube is important for preserving the recurrent laryngeal nerve and vagus nerve in thyroid surgeries with IONM. Injury of these nerves during surgery and resultant vocal cord paralysis results in several complications that worsen patients’ quality of life, from mild hoarseness to dysphagia, and even asphyxia and tracheostomy, which might result in medicolegal consequences^18^. For qualified intraoperative neuromonitoring in thyroid surgery, sufficient exposure of the vocal cords is important to place the surface electrodes of the EMG tube between the vocal cords precisely. Additionally, maintaining an adequate position of the tube during surgery is important for successful IONM.

Some previous studies have shown that the tube position was changed by patient position change, such as neck extension, flexion, and rotation^5,19–25^. A previous animal study directly showed that positional changes of the EMG tube inserted into the piglet’s trachea could change the amplitude of the EMG signal during neural monitoring^26^. The previous study showed that tube movement of 1 cm from the optimal position led to decrease of the amplitude by 500 to 600 μV approximately^26^. As a result, malposition of the tube might result in false loss of signal and increase the risk of RLN damage^4,27,28^. Therefore, tracheal intubation in the thyroid surgical position might be worth pursuing, both in terms of precise neuromonitoring and reduction in repetitive repositioning of the tube because that can reduce tube displacement due to position change of the patient for thyroid surgery. In addition, tube displacement due to position change of the patient can cause cuff pressure change, intraocular pressure change, or cardio-respiratory response^6,29,30^.

There might be concerns about deterioration of the glottic view during intubation in the thyroid surgical position. It is well known that video laryngoscopes improve the glottic view, even though it has been proven in the sniffing or neutral position, but not in the thyroid surgical position. A previous study showed that tube adjustment was necessary for qualified IONM in 66.5% of 297 patients who underwent thyroid surgery after surgical positioning following tracheal intubation with an EMG tube^31^. Another study reported the usefulness of the video laryngoscope in proper tube positioning for IONM during thyroid surgery^3^. However, investigators performed laryngoscopy twice to achieve successful placement of the EMG tube for IONM in the previous study. That is, they performed a second laryngoscopy to inspect for any alteration in tube placement after surgical positioning for thyroid surgery^3^. In the present study, we performed laryngoscopy once in the thyroid surgical position with a video laryngoscope to place the EMG tube correctly, with apneic time of 32.1 ± 6.1 s, which was acceptable, even if it took a little longer due to the observations required for the study.

A previous study reported that the first-attempt success rate with video laryngoscopy in proper tube positioning without tube position adjustment was 97.5% (39/40) with the patient’s neck extended^32^, even though the neck extension seemed to be different from the surgical position in our study. Although the first-attempt success rate of the study was similar to that of ours (100%), they did not present objectively the difficulty of tracheal intubation, such as the modified C–L grade or POGO scale. We hypothesized that video laryngoscopy might provide sufficient vocal cord exposure to appropriately place the EMG tube in the thyroid surgical position. In this study, the glottic view was also clearly improved in video laryngoscopy compared with direct laryngoscopy while the patients were in the thyroid surgical position. We investigated this using the POGO scale, which was the basic tool of the primary outcome in the study, because it is intuitive for evaluating the extent of the vocal cord exposure, and has good inter-physician/intra-physician reliability^15^, even though it is a subjective assessment as the modified C–L grade. The more the vocal cords can be exposed during laryngoscopy, the easier it is for clinicians to accurately position the EMG tube. Our study showed that point objectively with POGO scale increase in video laryngoscopy compared to direct laryngoscopy. Additionally, our results showed this in the modified C–L grade, which was graded as 1 or 2A with video laryngoscopy in most cases of the study. As a result, precise tracheal intubation of the EMG tubes for IONM was successfully achieved without external laryngeal manipulation in video laryngoscopy for all patients except one in the present study. On the contrary, the glottic view without external laryngeal manipulation during direct laryngoscopy deteriorated, with only 17 participants were graded as modified C–L grade 2A, and none as grade 1. Given that the thyroid surgical position is suboptimal for aligning airway axes, the deterioration in glottic exposure with direct laryngoscopy might be inevitable.

The advantages of video laryngoscopy are well known for clinicians by numerous studies^33^. From this, clinicians can easily guess video laryngoscopy might improve the glottic view during tracheal intubation in any patient position, including the thyroid surgical position. However, many studies have presented usefulness of video laryngoscope only in a position with the patient’s neck neutral rather than the sniffing position for patients with cervical spine instability^34^. We believe that performing tracheal intubation in the thyroid surgical position can prevent or minimize tube movement due to changing the patient position for thyroid surgery after tracheal intubation in cases where tracheal intubation is not performed in the thyroid surgical position. However, it seems that clinicians do not agree to perform tracheal intubation in the thyroid surgical position because it is well known that the sniffing position is optimal for tracheal intubation. This perception of clinicians who consider tracheal intubation to be very important does not seem to be much different even when video laryngoscopes have become widespread. In addition, we showed the relatively poor C–L grade in direct laryngoscopy during tracheal intubation in the thyroid surgical position^9,11^. However, it is basically important that the electrodes on the specialized tracheal tube contact with the vocal cords and do not move after confirmation of adequate positioning for qualified IONM. Therefore, we planned to perform this study to show usefulness of video laryngoscopy in positioning the EMG tube adequately during tracheal intubation in the thyroid surgical position. we hoped that clinicians try to perform tracheal intubation with a specialized tracheal tube, such as an EMG tube by a video laryngoscope in the thyroid surgical position from our results. However, it must be the highest valuable always to secure patient safety. That is, clinicians should suspect difficult airway before deciding which position of the patient for tracheal intubation because the sniffing position should be superior than any other patient position for tracheal intubation, even though all video laryngoscopy in the thyroid surgical position were graded as the modified C–L grade 1 or 2A, even without external laryngeal manipulation, in the present study. In difficult airway cases, it may be better to perform tracheal intubation in the sniffing position first and then assume the thyroid surgical position in thyroid surgery with IONM.

There were some limitations in the study. First, direct comparison between video laryngoscopy and direct laryngoscopy with a direct laryngoscope, such as a Macintosh blade was not performed for real tracheal intubation. We performed this study to compare video and direct laryngoscopy with a design of comparison within the same patient. That is, we performed tracheal intubation under video laryngoscopy just after the evaluation of both direct and video laryngoscopy sequentially, using the same McGRATH™ MAC video laryngoscope with a disposable Macintosh type blade because the glottic view in video laryngoscopy was superior. Therefore, other outcomes such as difficulty of intubation, or intubation time during real tracheal intubation practice could not be compared between two conditions in the study. Second, a comparison of video laryngoscopy between the sniffing position and surgical position for thyroid surgery was not performed in this study. In a previous study, video laryngoscopy facilitated the EMG tube placement in patients in sniffing position, compared to direct laryngoscopy^35^. In addition, two previous studies showed that the modified C–L grade was disadvantageous in the surgical position for thyroid surgery^9,11^, compared with that of a previous study in which tracheal intubation was performed in the sniffing position^36^. We think that the result of our study is in line with the results of the previous studies. Third, the quality of IONM was not assessed in the present study. We considered the improved glottic view as a surrogate for accurate EMG tube positioning, assuming that EMG tube placement would be more precise when the vocal cords are more exposed. During study period, moreover, additional adjustment of the tube insertion depth for optimization of IONM signal quality was rarely performed after the primary tracheal intubation and tube fixation in the study population, and the surgical team performed the surgery with satisfactory IONM in all cases. However, caution is required in interpreting the results, as the improved glottic view does not guarantee directly the quality of IONM. Further study regarding the usefulness of video laryngoscopy for improving the quality of IONM might be needed. Lastly, to avoid multiple laryngscopies, we utilized the McGRATH™ MAC video larygoscope as a direct laryngoscope instead of conventional Macintosh blades to obtain and evaluate direct laryngoscopy. This choice was made because the shape of McGRATH™ MAC blade is closely resembles that of the Macintosh blade. However, it is important to note that the glottic view achieved using the McGRATH™ MAC video laryngoscope as a direct laryngoscope might differ from that obtained with a real Macintosh laryngoscope^37^. The previous study showed that the Intubation Difficulty Scores with the McGRATH™ MAC as a direct laryngoscope was higher than those with the Macintosh laryngoscope and McGRATHTM MAC video laryngoscope. Therefore, caution is advised when interpreting the result of our study to the potential for differences in the direct laryngoscopy between with the McGRATH™ MAC video laryngoscope and the conventional Macintosh laryngoscope. However, the two previous studies showed the poor C–L grade in direct laryngoscopy during tracheal intubation in the thyroid surgical position^9,11^.

In conclusion, video laryngoscopy could improve the glottic view when compared to direct laryngoscopy in the thyroid surgical position. The utilization of video laryngoscopy, in conjunction with the thyroid surgical positioning before tracheal intubation, may contribute to avoiding the need for tube repositioning, thereby facilitating the achievement of qualified IONM during thyroid surgery.

## Data Availability

The datasets generated during and/or analyzed during the current study are available from the corresponding author on reasonable request.
